# Preventing the acute skin side effects in patients treated with radiotherapy for breast cancer: the use of corneometry in order to evaluate the protective effect of moisturizing creams

**DOI:** 10.1186/1748-717X-8-57

**Published:** 2013-03-12

**Authors:** Rossella Di Franco, Elena Sammarco, Maria Grazia Calvanese, Flora De Natale, Sara Falivene, Ada Di Lecce, Francesca Maria Giugliano, Paola Murino, Roberto Manzo, Salvatore Cappabianca, Paolo Muto, Vincenzo Ravo

**Affiliations:** 1 Dipartimento Assistenziale di Radiologia, Radioterapia, Medicina Nucleare -Seconda Università di Napoli, Napoli, Italy; 2 U.O.S. of Dermatology P.O. Ascalesi, Napoli, Italy; 3 U.O.C. of Radiotherapy INT "G. Pascale", Napoli, Italy; 4 U.O.C. of Radiotherapy P.O. Ascalesi, Via Egiziaca a Forcella 31, Napoli, 80131, Italy

**Keywords:** Breast cancer, Cutaneous damage, Topical treatment, Radiotherapy, Corneometry

## Abstract

**Background and purpose:**

The purpose of this study was to add, to the objective evaluation, an instrumental assessment of the skin damage induced by radiation therapy.

**Materials and methods:**

A group of 100 patients affected by breast cancer was recruited in the study over one year. Patients were divided into five groups of 20 patients. For each group it was prescribed a different topical treatment. The following products were used: Betaglucan, sodium hyaluronate (*Neoviderm®*), Vitis vinifera A. s-I-M.t-O.dij (*Ixoderm®*), Alga Atlantica plus Ethylbisiminomethylguaicolo and Manganese Cloruro (*Radioskin1®*) and Metal Esculetina plus Ginko Biloba and Aloe vera (*Radioskin 2®*); Natural triglycerides-fitosterols (*Xderit®*); Selectiose plus thermal water of Avene (*Trixera+®*). All hydrating creams were applied twice a day starting 15 days before and one month after treatment with radiations. Before and during treatment patients underwent weekly skin assessments and corneometry to evaluate the symptoms related to skin toxicity and state of hydration. Evaluation of acute cutaneous toxicity was defined according to the RTOG scale.

**Results:**

All patients completed radiotherapy; 72% of patients presented a G1 cutaneous toxicity, 18% developed a G2 cutaneous toxicity, 10% developed a G3 toxicity, no one presented G4 toxicity. The corneometry study confirmed the protective role of effective creams used in radiation therapy of breast cancer and showed its usefulness to identify radiation-induced dermatitis in a very early stage.

**Conclusions:**

The preventive use of topic products reduces the incidence of skin side effects in patients treated with radiotherapy for breast cancer. An instrumental evaluation of skin hydration can help the radiation oncologist to use strategies that prevent the onset of toxicity of high degree. All moisturizing creams used in this study were equally valid in the treatment of skin damage induced by radiotherapy.

## Introduction

The most important aspects in the management of radiation–induced skin reactions are prophylaxis and, when they appear, their appropriate treatment. Breast cancer has an high incidence rates in Italy, and it is the first cause of cancer death in women [1]. Screening programs increased the diagnosis of breast tumors in the initial stage and then survival [2]. Every year about 35,000 new cases are diagnosed and 80% of these being treated in Italian Radiotherapy Centers [3]. A great number of patients are submitted to adjuvant radiotherapy after surgery with or without chemotherapy. In the course of radiotherapy, skin toxicity remains an important clinical problem for many patients. The severity of the acute toxicity is related to several factors: dose per fraction, total delivered dose, location and volume of the treated area, radiating energy, other treatments received concomitantly or before. The literature shows individual variations depending on age, chronic diseases, skin types, genetic predisposition, skin damage from the previous and concomitant drug therapy (cytotoxic chemotherapy) [4,5].

However, it is difficult to estimate the frequency and intensity of these adverse effects. Temporary skin reactions vary from mild erythema to brisk moist desquamation. At present, guidelines regarding skin care during the course of radiotherapy remain inconsistent. Moreover all treatments remain linked to a subjective evaluation of the patient that the specialist involved (Dermatologist, Radiation Oncologist, Surgeon or Medical Oncologist) performs, in the majority of cases without a multidisciplinary evaluation [6].

Our treatment policies provide a prophylactic use of a topical therapy to prevent skin damages. The purpose of this study was to add to the subjective evaluation of the topical therapy, an instrumental measurement for the assessment of skin damage induced by radiation therapy.

## Methods and material

From January 2011 to December 2011 we enrolled a group of 100 consecutive female patients, aged from 29 to 75 years (median age 59) with a pathologic diagnosis of breast cancer. Were included in the study patients treated with conservative surgery (quadrantectomy), surgical margins were free of disease, and there wasn’t indication for regional nodal radiation therapy (less than four nodes involved) according to our national guidelines [7]. Therefore they were candidates for adjuvant radiotherapy with 6 MV photons, with a dose of 50 Gy (2Gy/fraction) to whole breast with tangential fields, and a subsequent additional dose of 10 Gy (2Gy/fraction) to the tumor bed [8]. All patients were submitted to a simulation Simulation CT-Scan, the three-dimensional treatment plan was set with the Pinnacle® TPS system, the target volumes were delineated according to the criteria of the International Commission on Radiation Units [9,10].

In our Department is active from 2010 a multidisciplinary ambulatory in which Dermatologists and Radiation Oncologists can visit the patients at the same time.

In Table 1 shows the characteristics of the patients.

**Table 1:** Characteristics of patients enrolled in the study

**Characteristics of patients**	**100**
**Phototype**	
I	0 (0%)
II	72 (72%)
III	28 (28%)
IV	0 (0%)
V	0 (0%)
**Skin hydration**	
dry skin	56 (56%)
normal skin	10 (10%)
sensible skin	34 (34%)

Seventy-two of these patients were of phototype II and 28 of phototype III according to the Fitzpatrick classification (Table 2) [11]; 56 patients had dry skin, 34 sensible skin and 10 normal skin.

**Table 2:** Human skin Phototypes (Fitzpatrick TB)


**I**	White	Always burn easily, never tans
**II**	White	Always burn easily, tans minimally and with difficulty
**III**	White	Burns minimally, gradually and uniformly
**IV**	Light brown	Burns minimally, always tans well
**V**	Brown	Rarely burns, tans profusely
**VI**	Dark brown or black	Never burns, tans profusely

Patients were randomized into five groups. Each group received the prescription of a prophylactic moisturizing cream.

Topical treatments prescribed to five groups were as follows:

**Group A** = Betaglucan, sodium hyaluronate (Neoviderm®)

**Group B** = Vitis vinifera A. s-I-M.t-O.dij (Ixoderm®)

**Group C** = Alga Atlantica and ethylbisiminomethylguaicolo Manganese Cloruro (Radioskin 1®) and metal esculetina, ginko biloba and Aloe vera (Radioskin 2®)

**Group D** = Natural triglycerides-fitosterols (Xderit®)

**Group E** = Selectiose, thermal water of Avene (Trixera+®)

Patients were evaluated, with corneometry, by dermatologists before and during radiotherapy and clinically together with radiation oncologists. At that moment of the CT Simulation they were instructed to apply cream topically every day (2–3 times/day) before (least 3 hours before) and after the radiotherapy treatment. They started radiation therapy medially 15 days after the beginning of the topical treatment and it was indicate to use the products also 1 month after the end of treatment. Moreover, during radiotherapy it was prohibited to use other types of creams or perfumes on the irradiated skin. Patients were asked not to apply the creams within 3 hours before radiation treatment and were educated to carefully wash the area only with special oil soap and to wear loose clothes, preferably made of cotton.

The patients were subjected to clinical examination plus Corneometry: 1) at the time of CT simulation, and 2) every week during treatment and 3) 1 month after the end of therapy. The evaluation was carried out jointly by the radiation oncologist and dermatologist. The radiation oncologist evaluated the radiodermatitis, according to the RTOG scale, the dermatologist evaluated the hydration of the skin with corneometry. The clinical examination consisted of physical examination of the breast skin, the corneometry measured numerically skin hydration. During treatment with radiotherapy, local side-effects were recorded according to the RTOG scale [12]. Corneometer CM® 820 is a simple instrument that measure the water content of the skin. The examination is based on the measure of the ability of a dielectric medium, so any change in the dielectric constant caused by the change of hydration of the skin alters the measurement supplied by the condenser [13]. The corneometry provides an indirect measure of the barrier function, and values of hydration of the skin are considered normal if included in the range 60–90 a.u.

## Results

Results were assessed not only in terms of grade and overall reduction in toxicity but also in terms of full regression of skin lesions with “ad integrum restitutio” and in terms of rapidity of repair of radio-induced damage assessed at 1 month after radiotherapy. At one month after the end of radiotherapy in all patients of groups A, B, C, E, it was recorded a G0 dermal toxicity. Eight patients in Group D had shown G1 toxicity. All patients completed the radiotherapy, and G4 cutaneous toxicity (according to RTOG scales (Table 3) was not observed in any patients [8].

**Table 3:** ROTG scale used

** Grade 0**	**Grade 1**	**Grade 2**	**Grade 3**	**Grade 4**
	Light and/or painless erythema	Sensitive and /or intense erythema	Desquamation	Ulceration
No changes	Epilation	Desquamation	Widespread sweating	Haemorrhage
	Desquamation	Partial sweating	Marked edema	Necrosis
	Dryness	Moderate edema		

The percentage of patients who underwent systemic therapies (chemotherapy, hormonal therapy and/or biological therapy) for each group are shown in Table 4. The complete results of the measurement with corneometry are shown in Table 5, that show the value of moisture of the skin measured with a corneometer. In general, corneometry values at starting point were between 40.5 (smallest median value) and 60.8 (highest median value) and at the end of treatment were between 45.7 (smallest median value) and 70.8 (highest median value). The low values of corneometry recorded at the beginning of treatment are certainly due to the alteration in skin of the breast treated surgically and any systemic treatments provided in specific cases.

**Table 4:** Percentage of patients treated with systemic therapies (chemo therapy CT, hormonal therapy OT and biological therapy BT) and breast volume for each group

	**CT %**	**OT%**	**BT%**	**Breast Volume > 500 cc**	**Breast Volume ≤ 500 cc**
**Group A**	50%	75%	14%	58%	42%
**Group B**	66%	66%	28%	60%	40%
**Group C**	40%	40%	20%	24%	76%
**Group D**	43%	85%	12%	74%	26%
**Group E**	60%	60%	25%	42%	58%

**Table 5:** Median of corneometry value at start and end of therapy

**Group A**	**Group B**	**Group C**	**Group D**	**Group E**
Start 40.6	Start 40.2	Start 42.6	Start 41.8	Start 40.7
(range 32.7 - 44.6)	(range 30.9 – 42.2)	(range 31.08 – 44.6)	(range 33.8 – 42.0)	(range 34.3 – 43.5)
End 70.8	End 75.7	End 65.8	End 70.9	End 70.9
(range 39.5 - 71.9)	(range 37.2 – 77.0)	(range 38.6 – 68.0)	(range 39.0 – 72.1)	(range 38.4 – 73.2)

We evaluated the correlation between the values measured with the corneometry in the various groups and systemic therapies carried out by the patients, in particular for each type of therapy (shown in Table 4) we evaluated the absolute risk reduction (ARR), relative risk (RR) and odds ratio (OR). We have not found a clear correlation between systemic treatments and the values of corneometry, probably for the small number of patients examined.

Regarding the correlation of the measurement with corneometry with the breast volumes, we found breast volumes > 500 cc both in groups B and D. We can hypothesize a correlation between breast volumes of the group D and skin toxicity, but without a statistical significance. The values of corneometry however were similar in these groups, so we can’t describe a direct correlation between the breast volumes, the corneometry values and the local toxicities.

### Group A

Of the 20 patients, 18 showed G1 toxicity and 2 G2 toxicity during radiotherapy. All patients had a good grade of hydration measured with a corneometer, also at the first follow up. Betaglucan and sodium hyaluronate encourage and speed up the regeneration and quickens the healing of irradiated skin. The level of skin elasticity and skin dryness is good.

### Group B

Of the 20 patients 16 showed G1 toxicity and 4 G2 toxicity during radiation therapy. Measurement of hydration showed a value of moisture of the skin with a slight decrease during the therapy and at the first follow up.

### Group C

Of these patients 19 manifested G1 and 1 G2 toxicity at the conclusion of radiotherapy. The measurement with a corneometer emphasized the effective role of moisturizing cream. The Alga Atlantica has a central role in protection the skin’s microcapillary integrity.

### Group D

Of 20 patients, 4 had G1 toxicity, 8 a G2 toxicity and 5 a G3 toxicity during the radiation treatment. Eight patients who suffered cutaneous toxicity during the treatment also showed G1 toxicity at the first follow up.

### Group E

Of the 20 patients, 15 had a G1 cutaneous toxicity, 5 a G2 toxicity. Selectiose has a role in inflammatory reactions. Avene thermal water and glycin modulate itch and reddening.

**Figure 1:**
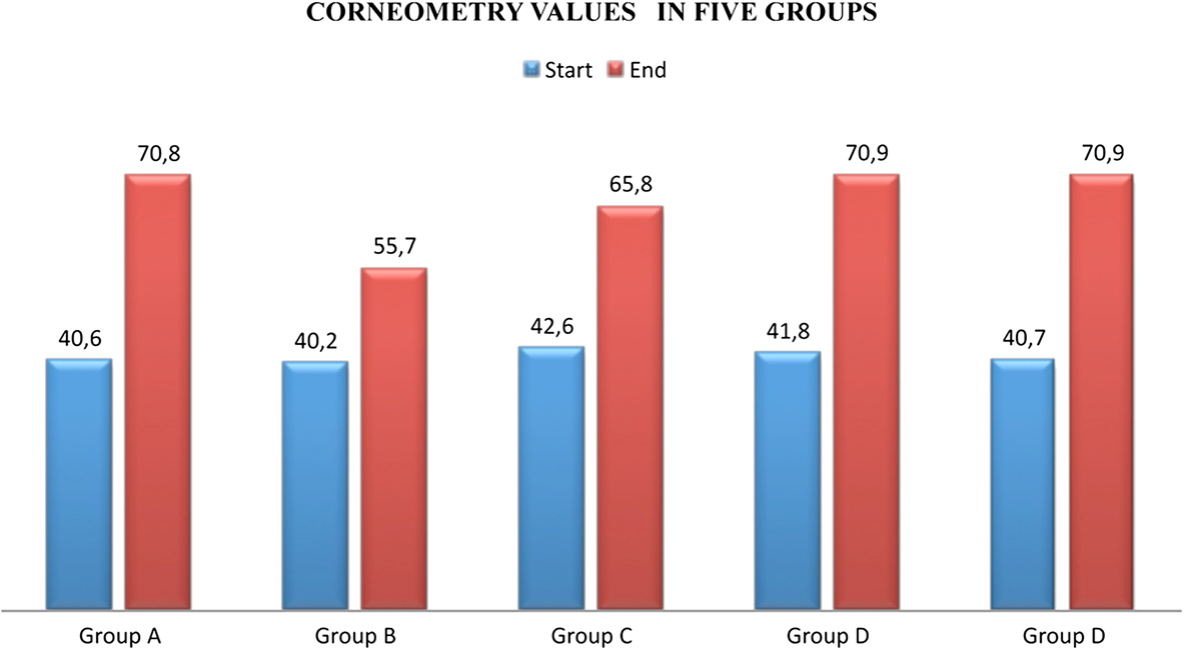
Mean Values of Corneometry at the start and the end of treatment.

**Figure 2:**
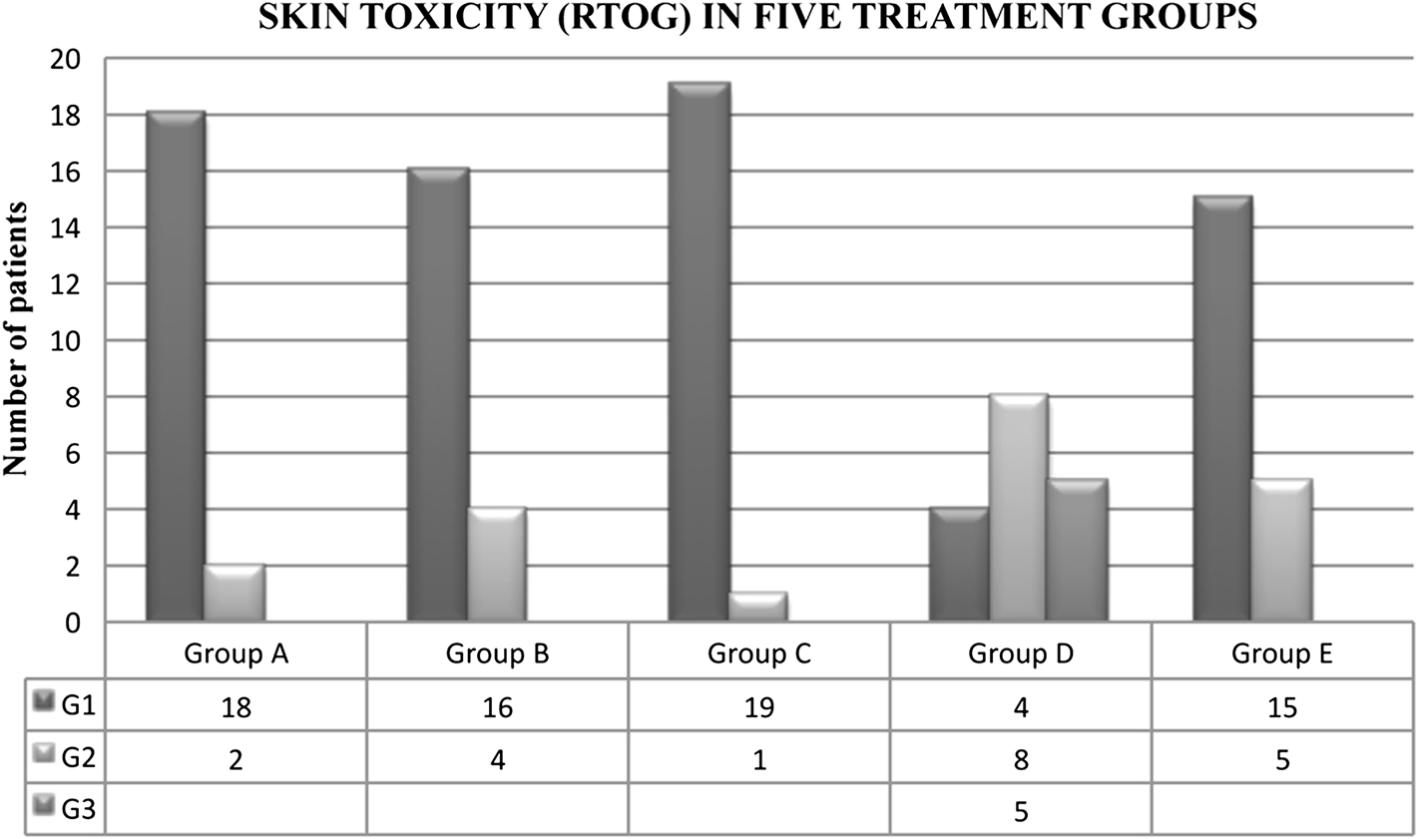
Skin toxicity observed in the five groups of patients.

The results concerning the average value of corneometry and dermal toxicity (RTOG), obtained in the five groups, are shown in the following graphics (Figure 1, Figure 2):

The measurement with a corneometer confirmed the protective role of effective creams used in our Departmets routinely during radiation therapy of breast cancer. This allowed us to identify radiation induced dermatitis in a very early stage compared to only objective examination by allowing to treat early stage patients with a greater hydration or with steroid creams to intervene with early onset of erythema.

## Discussion

Skin side-effects, using radiotherapy techniques, are a common consequence of radical doses of radiotherapy, and many products have been introduced in order to prevent such side effects. Now radiation techniques have been improved but concomitantly there is a wider attention on the problems linked to skin irradiation. Currently, there is no standard approach for the prevention and treatment of radio-induced skin lesions, although several studies have been published on the use of various kinds of topical agents [14,15]. Patients with breast cancer are increasing and their call for help expresses the need for new medical equipment. There is a growing attention on the local side effects of therapies in the field of oncology, and radiotherapy above all, also because there is an increasing request by these patients, to preserve as much as possible their physical aspect. Despite the common occurrence of radiation skin toxicity, there are very few trials on the argument.

In our study, we show that a good hydration of the skin before, during and after radiotherapy treatment can have a positive effect on the skin tolerance of treatment. In our clinical practice we use to prescribe moisturizers to all patients with an indication for radiotherapy for breast cancer. In fact we believe that a preventive therapy of the skin side effects is more effective than a treatment of the local side effects once they appear. This approach shows significant effectiveness in reducing the onset of acute dermal toxicity. Moreover, the radiotherapy treatments were well tolerated by patients and no treatment delays were necessary because of skin reactions. Acute radiodermatitis may affect the patients’ quality of life and daily activities. In some cases, unplanned gaps in treatment could occur, which decrease treatment efficacy [16]. Apart from thorough skin cleaning and prevention of local irradiation-induced trauma, there is no well established protocol to prevent skin radiation toxicity [17]. According to literature data we know that there are some risk factors including concurrent therapy, the use of bolus and smoking, previous or concomitant chemotherapy because of the change in the smallest blood vessels [4,5].

In our experience we tested five different products in one hundred patients. The formulations of creams used showed good results. For all of them we received a satisfactory result, with only five patients with a grade 3 dermatitis and twenty with a grade 2 dermatitis. It is impossible for us to draw a conclusion about the question of what product is more active also because the number of patients is too small and because there are too many variables in the groups. In fact there are personal differencies, of the phototypes, but also differencies about the age of patients and the therapies that they received before, such as chemotherapy, or after surgery and concomitantly with radiotherapy, like ormonal therapy. The largest number of patients with a recorded skin toxicity a month after radiotherapy, in group D of our study, can be related to the patient’s biggest breast volumes, although we do not have a statistical significance. Moreover the values of corneometry allow us to attribute a good moisturizing effect for products used in five groups of patients. Certainly the creation of a multidisciplinary team has allowed, with the corneometry conducted at the same time of the clinical evaluation, to identify cases of skin toxicity in a first phase and to determine the appropriate treatment to prevent the onset of dermatitis of high level which may affect the tolerance of treatment for these women.

The first physiopathological step of radiation toxicity to the skin is known to be an overproduction of free radicals, which is responsible for damage to the basal epidermis layer cells and the endothelial cells. Degradation of the latter cells induces a perivascular inflammatory infiltrate around dilated blood vessels. Degradation of the former cells (proliferative layer) induces the transcription of pro-inflammatory cytokines, which are responsible for the inflammatory cascade. As a consequence of those effects, early radiodermatities are clinically observed [18-20]. The physiopathology of moist desquamation involves the loss of basal layer cells of epidermis. Erythema reflects an inflammatory response and a damage to basal layer cells. Aloe vera could be used to limit erythema, leading to an overall lower skin toxicity score. In literature, the prophylactic or therapeutic use of topical betamethasone is often present. The evaluation of skin side effects during and after a radiotherapy treatment normally is a clinical evaluation and it represents a subjective point of view of the phenomenon. In our study, we have combined clinical examination with corneometry. Although it is a measure of the water content of the skin, it is only an indirect measure of barrier function. However, there is a good relationship between numeric value and the extent of hydration under various physiological and pathological phenomena. Since water loss through the skin normally occurs by passive diffusion through the epidermis, smaller values indicate greater water loss and are consistent with increased damage of the barrier function of the stratum corneum [21-23]. Use of Corneometer to study water content of the skin is valid because of the skin has mechanic properties in relationship to hydration state; so a dry skin will be less elastic than moisturized skin. The assessment “in vivo” of the hydration is very important for clinical and experimental evaluation of the real capacity of a cream to moisturize skin and repair skin barrier. Some patients started radiotherapy already with low hydration value probably because of the chemotherapy treatment received before radiotherapy. Women treated for breast cancer request a satisfactory aesthetic condition according to a resolution of oncologic problems. It was impossible for us with a small number of patients, to evaluate which is the best product of the five used.

## Conclusions

Our study confirms that moisturizers have an important value in the prevention of radiotherapy-induced skin damage and indicates that an instrumental assessment of skin hydration can help the radiation oncologist to use strategies that prevent the onset of toxicity of high degree.

Today it is ethically unacceptable to wait for the appearance of side effects to treat them. The prevention is required. Our patients receive a positive relationship with the radiation, which occurs after surgery and sometimes after chemotherapy, because they feel that the oncology team seeks to preserve their femininity. The interaction between medical specialties (Dermatology and Radiotherapy) has been valuable in this experience and has brought added value to the benefit of our patients.

## Competing interests

The authors declare that they have no competing interests.

## Authors’ contributions

ADL, MGC, SF, performed the literature search, extracted relevant articles and drafted the manuscript, VR, PM, PM, RM and SC contributed equally to this work participating in the design and coordination of the study, RDF, ES, FDN and FMG participated in the design of the study. All authors read and approved the final manuscript.

## References

[B1] Malvezzi M Bosetti C Negri E La Vecchia C Decarli A Cancer mortality in Italy,1970-2002 Tumori 2008 94 640 657 19112936 10.1177/030089160809400502

[B2] Gotzsche PC Nielsen M Screening for breast cancer with mammography Cochrane Database Syst Rev 2006 19 1 CD00187 10.1002/14651858.CD001877.pub2 17054145

[B3] Robello A Morra A Orsatti M Giannini M Girlando A Cesaro MG Borrelli D Ascione C Giugliano FM Nicolucci G La Radioterapia in Italia: Censimento delle strutture e delle attività 2008 AIRO; Ed. Graft Prints

[B4] Tucker S Turesson I Thames H Evidence of individual differences in the radiosensitivity of human skin Eur J Cancer 1992 28A 1782 1791 10.1016/0959-8049(92)90004-l 1389511

[B5] Porock D Krisjanson L Nikoletti S Cameron F Pedler P Predicting the severity of radiation skin reactions in women with breast cancer Oncol Nurs Forum 1998 25 1019 1029 9679261

[B6] Ravo V Calvanese MG Di Franco R Crisci V Murino P Manzo R Morra A Cammarota F Muto P Prevention of cutaneous damages induced by radiotherapy in breast cancer: an institutional experience Tumori 2011 97 732 736 22322839 10.1177/030089161109700609

[B7] Gruppo di Lavoro AIRO per la Patologia Mammaria *La Radioterapia dei Tumori della Mammella – Indicazioni e Criteri Guida* 2009 AIRO; Ed. Graft Prints

[B8] Special Issue Radiotherapy of breast cancer Radiother Oncol 2007 82 243 10.1016/j.radonc.2007.02.003 17316854

[B9] International Commission of Radiation Units and Measurements ICRU Report 50: Prescribing, recording, and reporting photon beam therapy 1993 Bethesda, MD: International Commission of Radiation Units and Measurements

[B10] Cox JD Stetz J Pajak TF *Toxicity criteria of the Radiation Therapy Oncology Group (RTOG) and the European Organization for Research and Treatment of Cancer (EORTC)* Int J Radiat Oncol Biol Phys 1995 31 5 1341 6 10.1016/0360-3016(95)00060-C 7713792

[B11] Fitzpatrick TB Eisen AZ Wolff K Freedberg IM Austen KF Dermatology in general medicine Mc Graw-Hill 1692 1695 1993

[B12] ICRU Report 62 Prescribing, recording, and reporting photon beam therapy (supplement to ICRU Report 50) 1999 Bethesda, MD: International Commission of Radiation Units and Measurements

[B13] Heinrich U Koop U Leneveu-Duchemin MC Osterrieder K Bielfeldt S Chkarnat C Degwert J Hantschel D Jaspers S Nissen HP Rohr M Schneider G Tronnier H Multicentre comparison of skin hydration in terms of physical-, physiological- and product-dependent parameters by the capacitive method (Corneometer CM 825) Int J Cosmet Sci 2003 25 45 51 10.1046/j.1467-2494.2003.00172.x 18494882

[B14] Schmuth M Wimmer MA Hofer S Sztankay A Weinlich G Linder DM Elias PM Fritsch PO Fritsch E Topical corticosteroid therapy for acute radiation dermatitis: a prospective, randomized, double-blind study Br J Dermatol 2002 146 6 983 991 10.1046/j.1365-2133.2002.04751.x 12072066

[B15] GozzoTde O Panobianco MS Clapis MJ de Almeida A Dermatological toxicity in women with breast cancer undergoing chemotherapy treatment Rev Lat Am Enfermagem 2010 18 4 681 687 20922313 10.1590/s0104-11692010000400004

[B16] Hansen O Overgaard J Hansen HS Overgaard M Hoyer M Jorgensen KE Bastholt L Berthelsen A Importance of overall treatment time for the outcome of radiotherapy of advanced head and neck carcinoma: dependency on tumour differentiation Radiother Oncol 1997 43 47 51 10.1016/S0167-8140(97)01904-X 9165136

[B17] Faithfull S Management of acute radiotherapy induced skin reactions: a literature review Eur J Oncol Nurs 2001 5 4 221 233 10.1054/ejon.2001.0145 12849619

[B18] Hymes SR Strom EA Fife C Radiation dermatitis: clinical presentation, pathophysiology, and treatment J Am Acad Dermatol 2006 54 28 46 10.1016/j.jaad.2005.08.054 16384753

[B19] Sitton E Early and late radiation-induced skin alterations. Part I: mechanism of skin changes Oncol Nurs Forum 1992 19 801 807 1608843

[B20] Roberts WE Skin type classification systems old and new Dermatologic Clinics 2009 27 529 533 19850202 10.1016/j.det.2009.08.006

[B21] Barel AO Clarys P Study of the stratum corneum barrier function by transepidermal water loss measurements: comparison between two commercial instruments: Evaporimeter and Tewameter Skin Pharmacol 1995 8 186 195 10.1159/000211345 7488395

[B22] Pinnagoda J Tupker R Coenraads PJ Nater JP Measurement of transepidermal water loss Nonivasive Methods and the Skin - (Serup, J. & Jemec, G. B. E.) 1995 Boca Raton, FL: CRC Press 173 178

[B23] Blichmann CW Serup J Assessment of skin moisture. Measurement of electrical conductance, capacitance and transepidermal water loss Acta Derm Venereol 1998 68 284 290 2459872

